# MicroRNA profiling of testicular Leydig cell tumors identifies a microRNA signature associated with malignancy and miR‐196b‐5p as a potentially useful biomarker

**DOI:** 10.1002/path.6487

**Published:** 2025-10-28

**Authors:** João Lobo, Nuno Tiago Tavares, Fernanda Fernandes‐Pontes, Vera Constâncio, Ana Teixeira Marques, Bruno Oliveira‐Lopes, Diana Fonseca, Carmen Jerónimo, Rui Henrique, Kvetoslava Michalova, Kristine M Cornejo, Maurizio Colecchia, Costantino Ricci, Muhammad T Idrees, Felix Contreras, Isabel M Fernandez Gonzalez, William J Anderson, Fiona MacLean, Adeboye O Osunkoya, Chia‐Sui Kao, Ankur R Sangoi, Thomas M Ulbright, Andres M Acosta

**Affiliations:** ^1^ Department of Pathology Portuguese Oncology Institute of Porto (IPO Porto)/Porto Comprehensive Cancer Center Raquel Seruca (P.CCC), R. Dr. António Bernardino de Almeida Porto Portugal; ^2^ Cancer Biology and Epigenetics Group, IPO Porto Research Center (GEBC CI‐IPOP), Portuguese Oncology Institute of Porto (IPO Porto)/Porto Comprehensive Cancer Center Raquel Seruca (P.CCC) & CI‐IPOP@RISE (Health Research Network), R. Dr. António Bernardino de Almeida Porto Portugal; ^3^ Department of Pathology and Molecular Immunology ICBAS ‐ School of Medicine and Biomedical Sciences University of Porto Porto Portugal; ^4^ Doctoral Programme in Biomedical Sciences ICBAS ‐ School of Medicine and Biomedical Sciences University of Porto Porto Portugal; ^5^ Department of Pathology Charles University, Faculty of Medicine in Plzen, Bioptical Laboratory, Ltd Pilsen Czech Republic; ^6^ Department of Pathology Massachusetts General Hospital, Harvard Medical School Boston MA USA; ^7^ Department of Pathology Vita‐Salute San Raffaele University, IRCCS San Raffaele Scientific Institute Milan Italy; ^8^ Pathology Unit, DIAP‐Dipartimento Interaziendale di anatomia patologica di Bologna Maggiore Hospital‐AUSL Bologna Bologna Italy; ^9^ Department of Pathology Indiana University Indianapolis IN USA; ^10^ Laboratorio de Patología Clínica Universitaria Unión Médica, PUCMM Santiago Dominican Republic; ^11^ Department of Pathology Brigham and Women's Hospital and Harvard Medical School Boston MA USA; ^12^ Douglass Hanly Moir Pathology Macquarie Park NSW Australia; ^13^ Department of Pathology and Urology Emory University School of Medicine Atlanta GA USA; ^14^ Department of Pathology and Laboratory Medicine Cleveland Clinic Foundation Cleveland OH USA; ^15^ Department of Pathology Stanford University Stanford CA USA

**Keywords:** Leydig cell tumors, sex cord stromal tumors, microRNAs, malignant, risk stratification, biomarkers

## Abstract

Approximately 10% of testicular Leydig cell tumors (LCTs) are clinically malignant and unresponsive to systemic treatment. Predicting their clinical behavior can be problematic because there are no biomarkers that can consistently discriminate between benign and malignant LCTs. We assessed microRNA expression profiles of LCTs to identify differentially expressed microRNAs that could potentially distinguish benign from malignant neoplasms. The study consisted of two phases. In the first (discovery) phase, we interrogated 768 microRNAs in a series of 11 LCTs (six malignant and five benign) using Taqman Low‐Density Array (TLDA) microRNA profiling. In the second phase, we validated the top differentially expressed microRNA targets with real‐time quantitative PCR on a series of 35 LCTs (17 malignant and 18 benign), assessing their clinical performance for distinguishing malignant from benign LCTs. Target biologic pathways were analyzed using the miRTargetLink 2.0 tool. A total of 50 microRNAs were differentially regulated in malignant LCTs (27 upregulated, 23 downregulated). The top six microRNA candidates (top three upregulated and top three downregulated) were validated, showing good performance for discriminating between malignant and benign LCTs, with an area under the curve (AUC) ranging between 0.69 and 0.87. MiR‐196b‐5p showed the best performance, with sensitivity, specificity, negative predictive value, positive predictive value, and accuracy of 82%, 83%, 83%, 82%, and 83%, respectively. A panel (i.e. combined) analysis reached 100% sensitivity and 83% specificity. Pathway analysis revealed significant overlap in the biological process targeted by the upregulated microRNAs in malignant LCTs, including proliferation, development, metabolism, hormone synthesis, and cell death. Our results support the idea that malignant LCTs are associated with a distinct microRNA signature. MiR‐196b‐5p was identified as a potentially useful biomarker to distinguish benign from malignant tumors. The shared downstream targets of the top upregulated microRNAs suggest that dysregulation of cell proliferation and apoptosis underlie aggressive biologic behavior in LCTs and may offer opportunities for targeted therapies. © 2025 The Author(s). *The Journal of Pathology* published by John Wiley & Sons Ltd on behalf of The Pathological Society of Great Britain and Ireland.

## Introduction

Leydig cell tumors (LCTs) are the most common sex cord stromal tumors of the testis, representing ~3% of adult and ~4%–9% of pediatric testicular tumors [1]. Most are benign and, therefore, cured by radical orchiectomy or testis‐sparing surgery with clear resection margins [2, 3]. Approximately 10% are clinically malignant as defined by the development of metastases; these neoplasms, like other malignant sex cord stromal tumors, are typically unresponsive to radiation and systemic treatment [4]. Patients with primary LCTs deemed potentially malignant may undergo upfront retroperitoneal lymph node resection [2, 5, 6]. This highlights the importance of proper patient selection for aggressive clinical management, especially considering that retroperitoneal lymph node dissection is associated with complications and morbidity [7]. However, prediction of malignant behavior in LCTs can be difficult and typically requires consideration of multiple clinicopathologic variables such as lymphovascular invasion, necrosis, tumor size, infiltrative pattern, cytologic atypia, mitotic index, proliferation index (Ki67), and patient age [8, 9, 10, 11, 12]. Evaluation of some of these parameters is somewhat subjective, and their weighted value as predictors of malignancy remains unknown.

Recent genomic studies have identified subsets of malignant LCTs characterized by fumarate hydratase (FH) deficiency (proposed to represent a distinct entity [13]), *Wnt* pathway activation, and aneuploidy/genomic instability [14]. Recurrent *MDM2* amplification and *TERT* fusion have also been described in clinically malignant (i.e. metastatic) LCTs, but neither are seen at very high frequencies (> 50%), nor have they been validated as biomarkers [8, 15, 16]. MicroRNAs have shown excellent performance as markers of testicular germ cell tumors, but their role in sex cord stromal tumors remains unexplored [17]. MicroRNAs are part of the family of noncoding RNAs that regulate gene expression at the posttranscriptional level, being typically deregulated in cancer [18]. In germ cell tumors, microRNAs of the 371–373 cluster are robust biomarkers of nonteratoma that were first discovered on array investigations [19], followed by validation on retrospective and prospective cohorts [20, 21]. We hypothesized that a similar approach might identify microRNAs useful to distinguish between benign and malignant LCTs.

In this study, we aimed to identify and validate a microRNA signature that discriminates between malignant and benign testicular LCTs. The study was designed in two phases: a discovery phase, in which we profiled 768 microRNAs using an array‐based platform, followed by a targeted validation phase, in which we assessed the top up‐ and downregulated microRNAs and evaluated their diagnostic performance.

## Methods

### Ethics approval and patient consent

Ethics approval for the study was obtained from the institutional review board of IPO Porto (CES1/018) and Indiana University (Protocol 18697, 2023). Patient consent was waived by national guidelines (i.e. only archival tissue was used).

### Samples

Malignant and benign LCTs were retrospectively retrieved from the pathology archives of the participating institutions. Selected slides were centrally reviewed and the diagnoses were confirmed by the senior author (AMA). Clinicopathological data were obtained from electronic medical records and pathology reports.

LCTs were defined as malignant/aggressive if they (1) showed evidence of metastatic disease and (2) showed ≥ 4 previously described risk factors, with at least one being necrosis and/or lymphovascular invasion [8, 9, 10, 12].

### RNA extraction and quality control

Representative formalin‐fixed paraffin‐embedded (FFPE) tumor blocks were selected, tumor foci were marked on hematoxylin and eosin (H&E) slides by a genitourinary pathologist, and the corresponding areas were dissected from 5‐μm unstained FFPE sections. Total RNA was extracted as previously described [22]. RNA concentration was measured using a Nanodrop Lite spectrophotometer (Thermo Fisher Scientific, Waltham, MA, USA), and RNA quality was evaluated on a Bioanalyzer 2100 Expert (Agilent Technologies, Santa Clara, CA, USA) using the Eukaryote Total RNA Pico assay (Agilent Technologies).

### Discovery phase: microRNA profiling

RNA from 11 LCTs (five clinically benign and six clinically malignant) was analyzed with the TaqMan Low‐Density Array (TLDA), using a previously described protocol [23]. Of the six malignant cases, five developed metastatic disease and one was a primary tumor with five risk factors, including extensive lymphovascular invasion. In brief, 1,000 ng total RNA was reverse transcribed using TaqMan MicroRNA Reverse Transcription Kit (Applied Biosystems, Waltham, MA, USA) and Megaplex™ Pools A and B RT primers (Thermo Fisher Scientific). cDNA (6 μl) was mixed with 450 μl TaqMan Universal PCR Master Mix (Thermo Fisher Scientific) and 444 μl of nuclease‐free water and loaded onto TaqMan™ Array Human MicroRNA A + B Cards Set version 3.0 (Thermo Fisher Scientific), which interrogated a total of 768 microRNAs. The reactions were performed using the QuantStudio 12K Flex Real‐Time PCR System with the appropriate array block (Thermo Fisher Scientific).

MicroRNA levels were analyzed using the Relative Quantification Application Module on the Thermo Fisher Cloud (Thermo Fisher Scientific). MicroRNAs with a cycle threshold (Ct) value over 35 were considered negative. MicroRNA levels were calculated using the 2^−∆∆Ct^ method [24], and array data were plotted using the SRplot web platform (https://www.bioinformatics.com.cn/en; last accessed 12 September 2025) [25]. A combined score composed of the sum of the *p* value (threshold 0.05) and fold‐change (threshold of 2) was used to identify differentially regulated microRNAs and select the top six (three upregulated and three downregulated) for validation. Differentially expressed microRNAs were plotted using a volcano plot, heatmap (unsupervised clustering), and principal component analysis (PCA), generated using the SRplot web platform [25]. T‐distributed stochastic neighbor embedding (*t*‐SNE) and Uniform Manifold Approximation and Projection (UMAP) were performed using the Rtsne and umap packages in base R (https://cran.r-project.org/web/packages/umap/index.html; https://cran.r-project.org/web/packages/Rtsne/index.html).

Furthermore, microRNAs of the miR‐371‐373 cluster in the LCTs were evaluated individually using data extracted from the arrays, analyzed, and plotted as previously described [26, 27].

### Validation phase: reverse transcription quantitative PCR (RT‐qPCR) for microRNA targets

Validation of the top six microRNA candidates selected was performed on a larger multi‐institutional series of LCTs (*n* = 35, 18 benign and 17 malignant). In brief, 100 ng total RNA was reverse transcribed using the TaqMan MicroRNA Reverse Transcription Kit (Applied Biosystems), and RT‐qPCR was performed on the QuantStudio 12K Flex platform (Applied Biosystems) using the Xpert Fast Probe (GRiSP, Porto, Portugal). TaqMan™ microRNA assays were used for hsa‐miR‐145‐5p (assay ID 002278, Applied Biosystems), hsa‐miR‐181c‐5p (assay ID 000482, Applied Biosystems), hsa‐miR‐182‐5p (assay ID 002334, Applied Biosystems), hsa‐miR‐196b‐5p (assay ID 002215, Applied Biosystems), hsa‐miR‐199a‐3p (assay ID 002304, Applied Biosystems), and hsa‐miR‐214‐3p (assay ID 002306, Applied Biosystems). RNU48 (assay ID 001006, Applied Biosystems) was used for normalization and endogenous quality control. Samples outside the expected amplification range were discarded due to poor quality [28]. All reactions were run in triplicate. MicroRNA levels were calculated using the 2^−∆∆Ct^ method. No template control (NTC) and no cDNA control were included. The data were analyzed on GraphPad Prism 10 (GraphPad Software, Boston, MA, USA) and presented as median and interquartile range. The nonparametric Mann–Whitney *U* test was used to compare microRNA levels between groups.

### Diagnostic performance of the validated microRNA candidates

The diagnostic performance of each microRNA for discriminating between malignant and benign LCTs was assessed with receiver operating characteristic (ROC) curves and the corresponding area under the curve (AUC). Biomarker performance metrics (sensitivity, specificity, positive predictive value, negative predictive value, and accuracy) were described using Youden's index [29] computed with the SPSS 29.0.1.1 software (IBM, Armonk, NY, USA). MicroRNA panel biomarker analysis was performed, using the pROC package in R (https://cran.r-project.org/web/packages/pROC/index.html).

Moreover, a logistic regression model was created to estimate the association between each microRNA level and benign/malignant or metastatic/nonmetastatic status, and the corresponding odds ratios (ORs) with 95% confidence interval (CI) and their respective *p* values were computed.

### Pathway analysis

Analysis of the target pathways of the top differentially regulated microRNAs was performed using data from miRTargetLink 2.0 (https://ccb-compute.cs.uni-saarland.de/mirtargetlink2; last accessed 12 September 2025) [30], which offers predictions of microRNA‐mRNA interactions using a comprehensive set of validated (miRTarBase) and predicted (miRDIP and miRDB) target databases. The leading pathways were plotted using the SRplot web platform [25].

### RT‐qPCR quantification of messenger RNA levels

Expression of selected targets identified on the pathway analysis was assessed with RT‐qPCR. Specifically, intracellular messenger RNA (mRNA) levels for *GATA6* (forward primer: GCCACTACCTGTGCAACGCCT; reverse primer: CAATCCAAGCCGCCGTGATGAA), *MKI67* (forward primer: TGACCCTGATGAGAAAGCTCAA; reverse primer: CCCTGAGCAACACTGTCTTTT), and β‐*GUS* (forward primer: ATGCCATCGTGTGGGTGAAT; reverse primer: CGGACCAGGTTGCTGATGTC) were quantified. The protocol was performed as previously described, with β‐*GUS* expression used for normalization [31].

## Results

### Clinicopathological features

**Figure 1:**
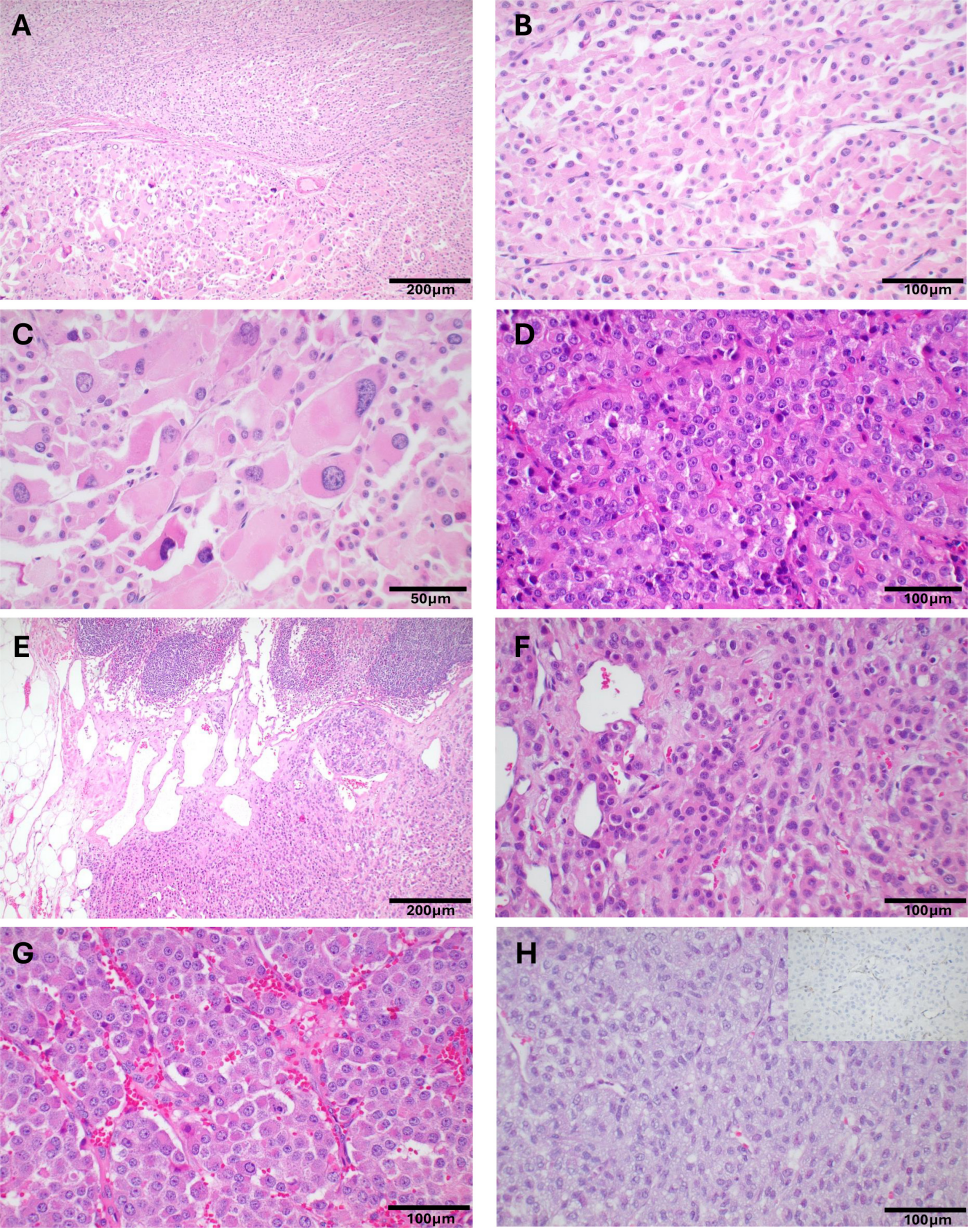
Histopathological features of malignant cases in the study cohort. (A–C) This LCT showed an abrupt transition between low and high‐grade areas (A). A low‐grade area with typical morphology is highlighted in panel B, whereas foci with high‐grade histology characterized by marked nuclear pleomorphism are highlighted in panel C. (D) Clinically malignant LCT without overt cytologic atypia. (E and F) LCT with infiltrative growth into hilar adipose tissue. (G) LCT with abundant mitotic figures and scattered apoptotic cells. (H) FH‐deficient LCT (FH immunohistochemistry is shown in the upper right inset).

A total of 35 testicular LCTs were included in the study, 17 malignant/aggressive and 18 benign. Of the 17 malignant/aggressive, 12 were proven metastatic and five showed at least four risk factors, one of them being necrosis and/or lymphovascular invasion. The median age at diagnosis for the malignant cases was 64 years (range 53–82 years). Seven cases were metastatic to retroperitoneal lymph nodes. Other sites of metastases included pelvis, abdominal wall, mesentery, peritoneum, urachus, lung, liver, and mediastinum. Images of representative malignant cases are presented in Figure 1, and a summary of their clinicopathologic features is provided in the supplementary material, Table S1.

### Discovery phase: microRNA profiling

**Figure 2:**
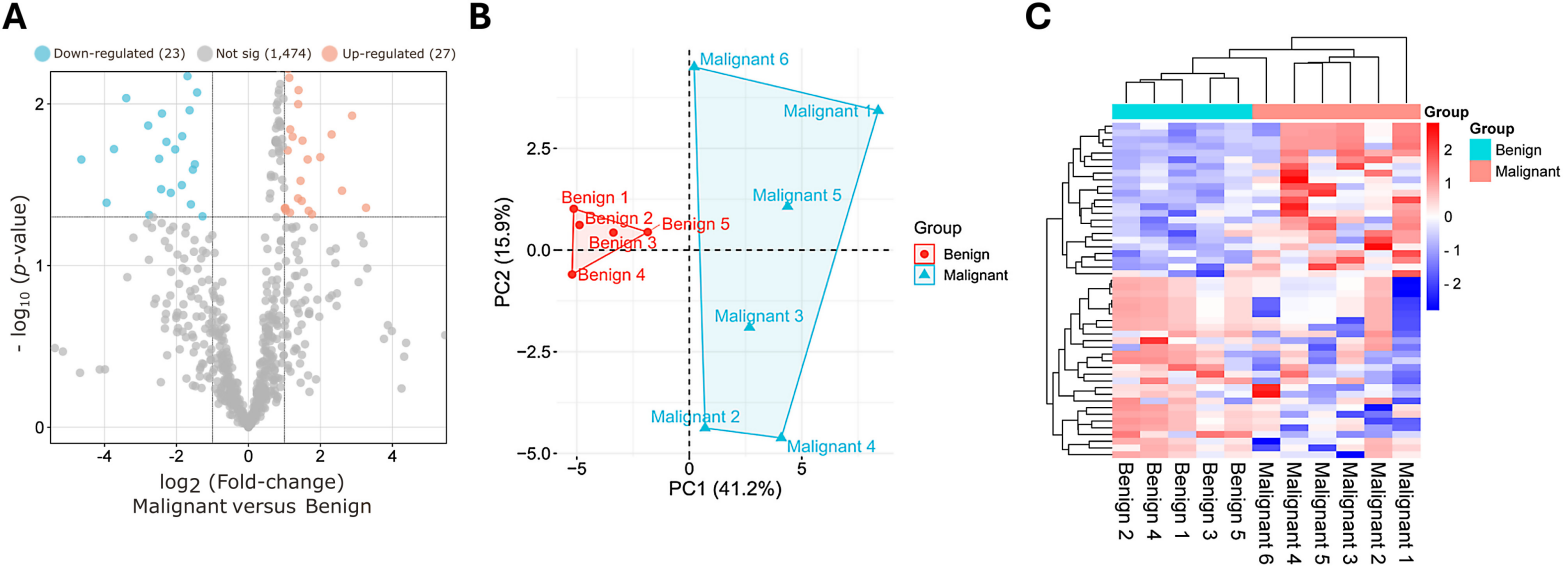
MicroRNA profiling of malignant versus benign LCTs. (A) Volcano plot showing differentially expressed microRNAs in malignant versus benign LCTs. (B and C) PCA and unsupervised clustering heatmap based on differentially expressed microRNAs that reached statistical significance.

Array‐based microRNA profiling (*n* = 768 microRNAs) identified a total of 50 microRNAs with statistically significant differential expression in malignant LCTs: 27 upregulated and 23 downregulated (Figure 2A). This microRNA signature discriminated malignant from benign LCTs using PCA and unsupervised clustering analyses, with clear separation of the two groups (Figure 2B,C). UMAP and *t*‐SNE analysis revealed similar findings (supplementary material, Figure S2). Of note, microRNAs of the 371–373cluster were negative in all LCT samples, highlighting their specificity for distinguishing nonteratomatous germ cell tumors from sex cord stromal tumors (supplementary material, Figure S1).

### Selection of microRNA candidates for validation

The full list of microRNAs analyzed is provided in the supplementary material, Table S3, while the list of differentially expressed microRNAs (*n* = 50) in malignant LCTs is provided in the supplementary material, Table S4. Based on the combined score described previously (supplementary material, Tables S5 and S6), the top three upregulated microRNAs selected were miR‐181c‐5p, miR‐182b‐5p, and miR‐196b‐5p, whereas the top three downregulated microRNAs selected were miR‐145‐5p, miR‐199a‐3p, and miR‐214‐3p.

### Validation phase and diagnostic performance of microRNA candidates

**Figure 3:**
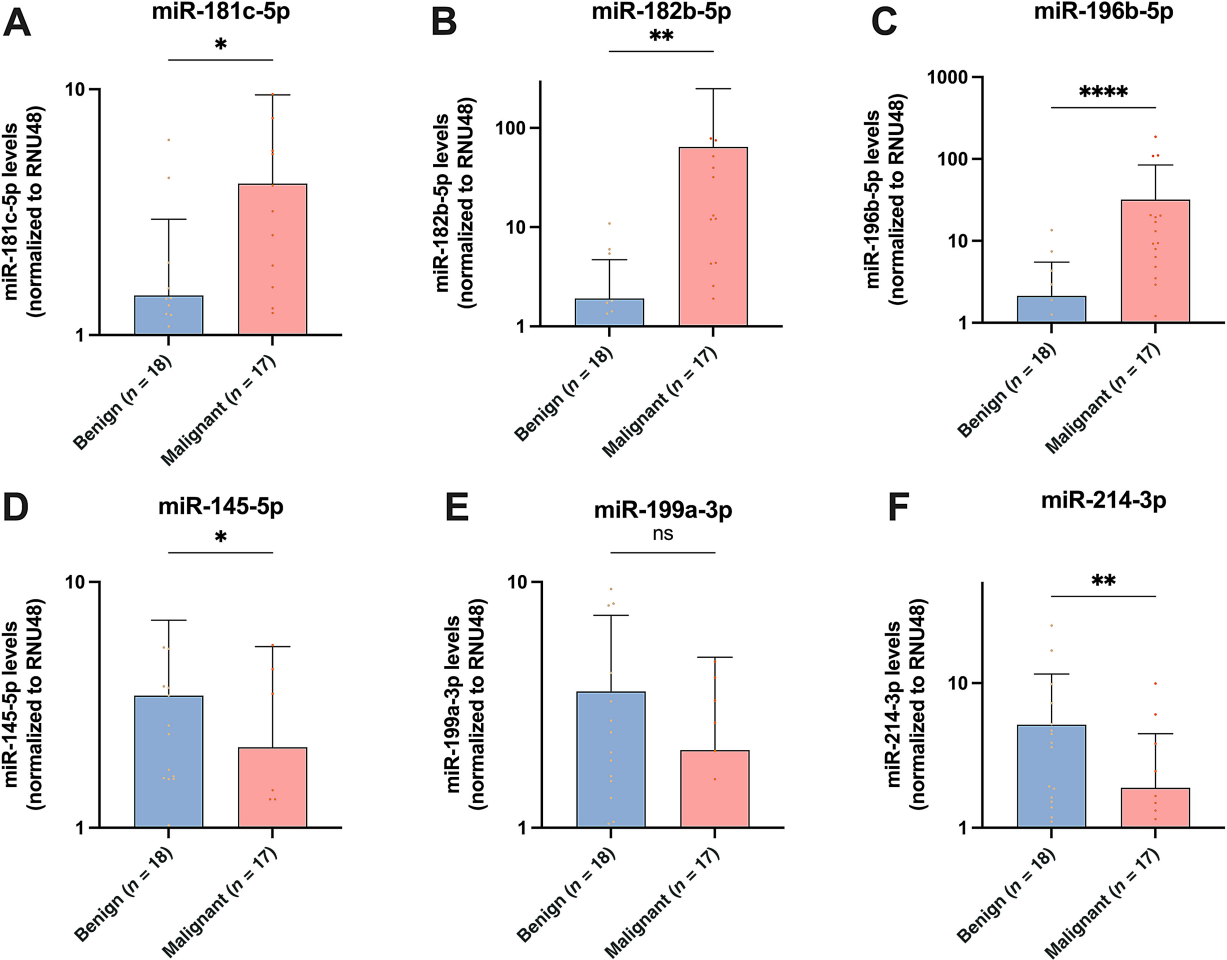
Validation of selected microRNA candidates in discriminating malignant LCTs. (A) miR‐181c‐5p, (B) miR‐182b‐5p, (C) miR‐196b‐5p, (D) miR‐145‐5p, (E) miR‐199a‐3p, and (F) miR‐214‐3p. Results are normalized to RNU48. *p < 0.05, **p < 0.01, ****p < 0.0001, ns denotes *p* > 0.05 (nonsignificant).

**Figure 4:**
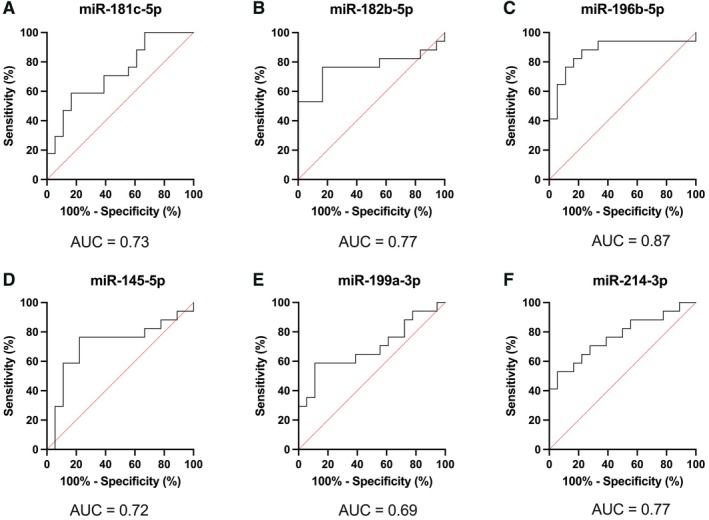
ROC curves for microRNA candidates in discriminating malignant LCTs. (A) miR‐181c‐5p, (B) miR‐182b‐5p, (C) miR‐196b‐5p, (D) miR‐145‐5p, (E) miR‐199a‐3p, and (F) miR‐214‐3p.

In the validation cohort (*n* = 35), the three candidate upregulated microRNAs showed significantly higher levels in malignant LCTs (*p* = 0.0435 for miR‐181c‐5p, *p* = 0.0143 for miR‐182b‐5p, and *p* < 0.0001 for miR‐196b‐5p; Figure 3A–C). The ROC curve and AUC of miR‐196b‐5p, miR‐182b‐5p, and miR‐181c‐5p are presented in Figure 4A–C and in Table 1. The three microRNAs discriminated malignant LCTs with AUC of 0.87, 0.77 and 0.73, respectively (Figure 4A–C). Of note, miR‐196b‐5p showed the best overall performance, identifying malignant LCTs with 82% sensitivity and 83% specificity. Positive predictive value, negative predictive value, and diagnostic accuracy were 82%, 83%, and 83%, respectively (Table 1).

**Table 1:** Biomarker performance parameters for discriminating malignant from benign LCTs.

microRNA	AUC	Sensitivity (%)	Specificity (%)	Positive predictive value (%)	Negative predictive value (%)	Accuracy (%)
miR‐181c‐5p	0.726	70.6	61.1	63.2	68.8	65.7
miR‐182b‐5p	0.765	76.5	83.3	81.3	79	80
miR‐196b‐5p	0.873	82.4	83.3	82.4	83.3	82.9
miR‐145‐5p	0.716	76.5	77.8	76.5	77.7	77.1
miR‐199a‐3p	0.693	64.7	61.1	61.1	64.7	62.9
miR‐214‐3p	0,771	70.6	72.2	70.6	72.2	71.4

Levels of two of the three candidate downregulated microRNAs (miR‐145‐5p and miR‐214‐3p) were significantly lower in malignant LCTs (*p* = 0.0293 and *p* = 0.0054, respectively; Figure 3D,F). The same tendency was found for the third candidate downregulated microRNA, miR‐199a‐3p, although the difference did not reach statistical significance (Figure 3E). The ROC curve and AUC for miR‐145‐5p, miR‐199a‐3p, and miR‐214‐3p are presented in Figure 4D–F and in Table 1. The three downregulated microRNAs showed AUCs of 0.72, 0.69, and 0.77, respectively (Figure 4D–F). Of these, the best discriminative performance was achieved by miR‐214‐3p, achieving an AUC of 0.77, with a sensitivity of 71%, specificity of 72%, positive predictive value of 71%, negative predictive value of 72%, and accuracy of 71% (Table 1). A biomarker performance analysis was also performed for the panel of microRNAs (supplementary material, Table S2). In this assessment, a combination of any three ‘positive’ microRNA results (i.e. above the cutoff for upregulated microRNAs and below the cutoff for downregulated microRNAs) yielded the best diagnostic performance, with a sensitivity of 100%, a specificity of 83%, and an AUC of 0.917. This was superior to the performance of miR‐196b‐5p alone.

**Figure 5:**
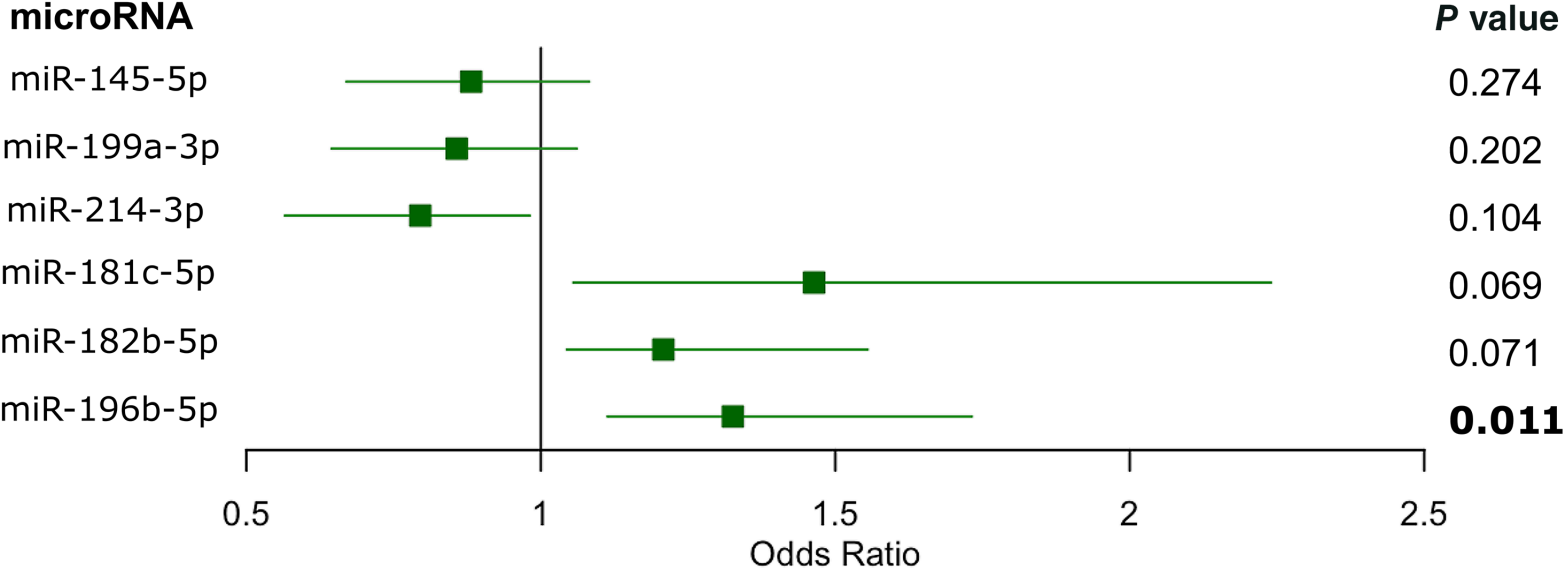
Forest plot representing the association of microRNA levels and the odds of malignant behavior. Odds ratio (OR), with 95% confidence interval and respective *p* values. Significant *p* value is represented in bold.

When computing a logistic regression model to calculate ORs, patients with higher levels of all three upregulated microRNAs showed increased odds of malignancy (OR = 1.46, 1.21, and 1.33 for miR‐181c‐5p, miR‐182b‐5p, and miR‐196b‐5p, respectively), with miR‐196b‐5p being statistically significant (*p* = 0.011). Additionally, patients with higher levels of the three downregulated microRNAs showed lower odds of being malignant (OR = 0.88, 0.86, and 0.80 for miR‐145‐5p, miR‐199a‐3p, and miR‐214‐3p, respectively) (Figure 5).

### Pathway analysis

**Figure 6:**
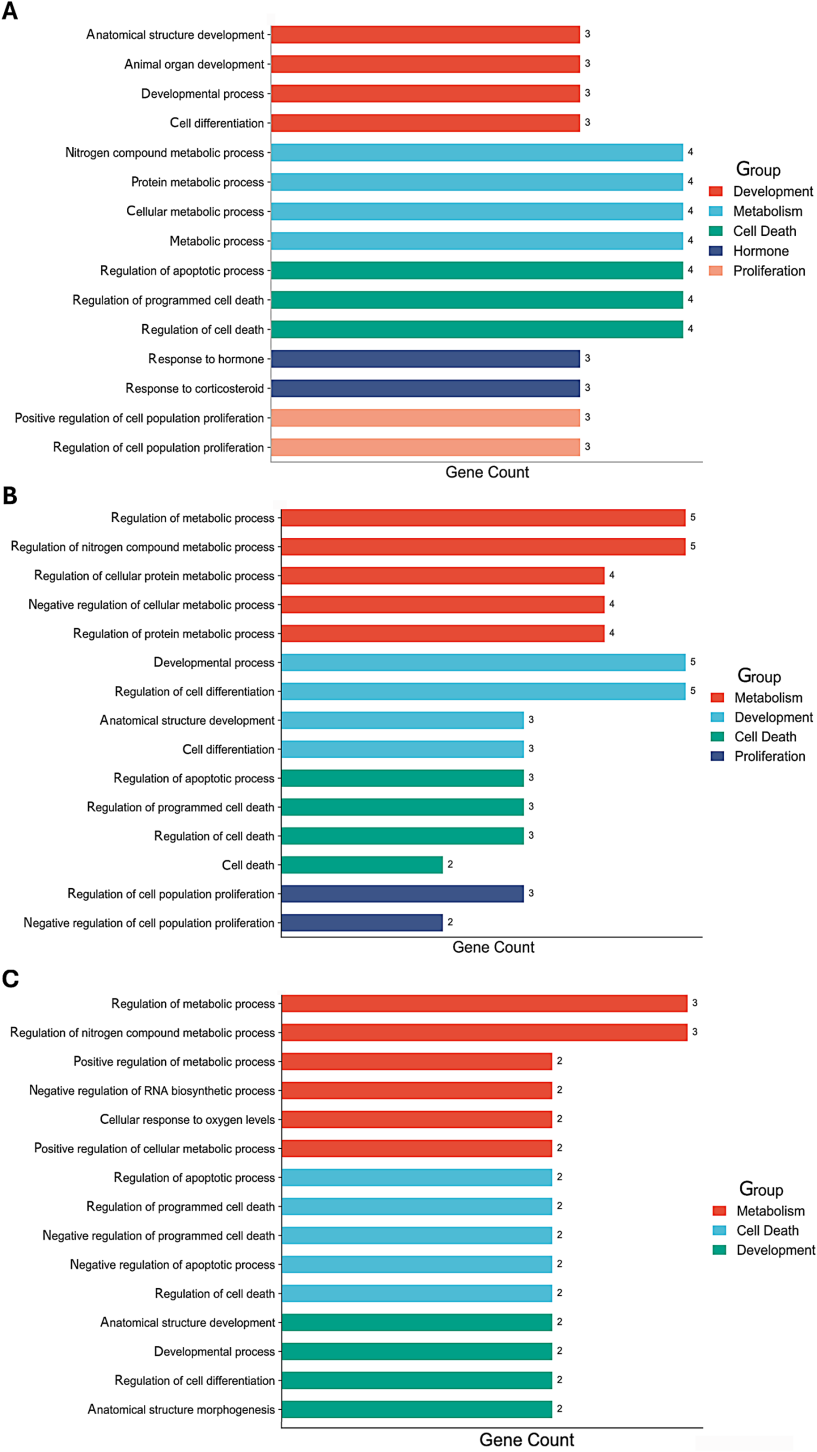
Pathway analysis for the validated upregulated microRNAs. (A) miR‐181c‐5p, (B) miR‐182b‐5p, and (C) miR‐196b‐5p.

The list of pathways and putative targets regulated by miR‐181c‐5p, miR‐182b‐5p, and miR‐196b‐5p is provided in the supplementary material, Tables S7–S12. Evaluation of pathways with two or more targets revealed marked similarities in processes regulated by these three microRNAs: development, metabolism, proliferation, cell death, and hormone synthesis (Figure 6A–C). Relevant targets within these pathways/processes included *KRAS*, *BCL2*, *GATA6*, *TRIM2*, *MITF*, *FOXO3*, *BDNF*, *RECK*, *MTSS1*, *PDCD4*, *HOXB8*, and *FAS*. To validate the predicted interactions of the differentially regulated miRNAs, two selected downstream targets, *GATA6* and *MKI67*, were further assessed with RT‐qPCR. As predicted using pathway analysis, malignant LCTs showed upregulation of *MKI67* and downregulation of *GATA6* (supplementary material, Figure S3).

## Discussion

LCT, the most common sex cord‐stromal tumor in men, shows a biphasic incidence with peaks in prepubertal children (between 5 and 10 years of age) and middle‐aged to elderly adults in the fourth to seventh decade of life [10, 32]. Children typically present with isosexual precocious pseudopuberty and/or gynecomastia, whereas most adult patients present with a painless testicular mass [32]. LCTs with typical morphology are easily recognized, but those with unusual features often require ancillary studies to be distinguished from other sex cord‐stromal tumors, germ cell tumors, or carcinoma [33, 34]. From a practical perspective, one of the diagnostic challenges in patients with LCT is to determine the risk of malignant behavior. Moreover, malignant LCTs are problematic because they are consistently resistant to systemic therapy [4]. A few multiparametric systems to determine risk of malignant behavior have been proposed in retrospective studies and meta‐analyses, but the weighted value of individual parameters has not been assessed, and some of them are subject to interpretation [8, 9]. Improvements in prognostication may be helpful for deciding the best management strategy, including consideration of upfront retroperitoneal lymph node dissection in selected high‐risk patients. Predictive biomarkers that are easy to assess in clinical practice would be ideally suited for this purpose.

Only a few studies have compared the molecular features of indolent and aggressive LCTs; hence, the number of candidate prognostic biomarkers is limited. Analyses of 25 LCTs with fluorescence *in situ* hybridization and comparative genomic hybridization have demonstrated recurrent chromosomal imbalances, including gains of chromosomes X, 19, and 19p and losses of chromosomes 8 and 16, but no clinically malignant (i.e. metastatic) neoplasms were assessed in this study [35]. More recently, two separate series of clinically malignant tumors identified recurrent *MDM2*/*CDK4* amplification in LCTs [8, 15], which correlates with MDM2 and CDK4 overexpression assessed by immunohistochemistry. Rizzo *et al* analyzed a cohort of 26 LCTs (18 clinically malignant or histologically aggressive) using next‐generation sequencing and immunohistochemistry, finding enrichment for *FH* alterations among malignant/aggressive neoplasms [14]. Hence, *MDM2* and *FH* may represent useful biomarkers, but they only identify a limited subset of malignant LCTs (< 50%) [36].

MicroRNAs are small noncoding RNAs capable of regulating gene expression post‐transcriptionally [37]. Their synthesis and secretion are typically dysregulated in cancer, contributing to tumor growth, progression, and aggressiveness. The impact of microRNAs on cancer biology has been remarkable, as highlighted by the recent awarding of the Nobel Prize in Physiology and Medicine to the investigators that discovered them [38]. Their small size, short half‐life, and high stability in circulation make them ideal candidate biomarkers that can be detected by fast, universal, cost‐effective, and user‐friendly quantitative techniques (such as RT‐qPCR) in liquid biopsies and tissues [39]. In this study of 35 testicular LCTs (17 malignant, 18 benign), we identified and validated a microRNA signature that discriminates benign from malignant tumors. Specifically, microRNA profiling of a discovery cohort detected 50 differentially regulated microRNAs in aggressive LCTs. Of note, microRNA profiles of aggressive LCTs demonstrated a higher degree of heterogeneity than those of benign counterparts (Figure 2), including a relative outlier (‘Malignant #6’), which nonetheless clustered with the remaining malignant cases. These variations possibly reflect (at least in part) a more heterogeneous genomic background of malignant cases [14, 15, 16]. None of the LCTs expressed microRNAs of the 371–373 cluster, a sensitive and specific biomarker for nonteratomatous germ cell tumors, a result that is consistent with prior investigations [40, 41]. Instead, we found that miR‐181c‐5p, miR‐182b‐5p, and miR‐196b‐5p were the top three upregulated microRNAs in malignant LCTs, demonstrating that they could discriminate between benign and malignant tumors with an AUC over 0.70 in validation studies. Two of the three top downregulated microRNAs also showed significantly lower levels in malignant LCTs in the validation phase, with miR‐214‐3p achieving the best performance, with an AUC of 0.77. Among individual microRNAs, miR‐196b‐5p showed the best diagnostic performance, with good specificity (83%), sensitivity (82%), and an overall accuracy of 83%. Additionally, patients with higher levels of this microRNA showed significantly higher odds of having malignant behavior (OR = 1.33, *p* = 0.0107). Panel analysis revealed that the combination of any three ‘positive’ microRNA results (i.e. above the cutoff for upregulated microRNAs and below the cutoff for downregulated microRNAs) yielded the best diagnostic performance: AUC = 0.917, sensitivity = 100%, and specificity = 83%. Pending external validation, the microRNA signature identified herein may be useful for identifying primary LCTs with malignant potential and represents a valuable addition to multiparametric systems. Importantly, considering the rarity of this entity, the cohort presented here includes a relatively large number of malignant LCTs from several institutions (*n* = 17). Moreover, stringent criteria were used to define LCTs as malignant (i.e. the presence of proven metastatic disease or ≥4 risk factors, with at least one of them being necrosis and/or lymphovascular invasion).

Assessment of biologic pathways and processes predicted to be impacted by the three top upregulated microRNAs demonstrated significant overlap, suggesting that these microRNAs may act synergistically to promote tumor growth and progression. One of the main affected processes is proliferation, consistent with prior reports of higher Ki67 indexes and higher mitotic counts in aggressive LCTs, and supported by the higher levels of MKI67 transcripts in malignant cases identified in the present study [12]. Another target biologic process is negative regulation of apoptosis, a hallmark of cancer that may explain in part the almost invariable resistance of malignant LCTs to systemic therapy [42]. Regulation of pathways related to metabolism by these microRNAs may reflect the degree of oxygen dependence of aggressive tumors and may be associated with the inclusion of a FH‐deficient LCT in the series (case ‘Malignant #9’), given the impact that inactivation of *FH* has on the Krebs cycle [43]. Finally, interference with pathways implicated in development and hormone synthesis aligns with the secretory function of Leydig cells and their role in the development of the male phenotype through the synthesis of steroid sex hormones (steroidogenic pathway) [44]. Regulation of developmental pathways by these microRNAs may indicate that LCTs show a more immature phenotype than nonneoplastic Leydig cells of the postpubertal testis [45]. *GATA6*, a transcription factor of the zinc finger pathway implicated in differentiation and regulation of epithelial‐to‐mesenchymal transition, was a common target of the top two upregulated microRNAs: miR‐181c‐5p and miR‐196b‐5p. Higher *GATA6* expression levels have been associated with favorable prognosis in gastric cancer [46]. In colorectal cancer, upregulation of miR‐196b‐5p was found to target and reduce the expression of GATA6, thereby acting as a positive regulator of the *Wnt*/beta‐catenin pathway in this context [47]. Upregulation of miR‐181 in hepatocellular carcinoma cancer stem cells with aggressive features led to the downregulation of GATA6, which is implicated in hepatocellular differentiation [48]. *In vitro* studies are needed to further explore the role of the miR‐196/miR‐181–GATA6 axis in LCTs and its possible links to the *Wnt* pathway, considering that some LCTs that show nuclear expression of beta‐catenin do not harbor hotspot *CTNNB1* mutations [49]. In our preliminary analysis, malignant LCTs showed lower levels of *GATA6* transcripts than benign LCTs, supporting this hypothesis.

In conclusion, we identified a microRNA signature that discriminated malignant from benign LCTs, validating a panel of upregulated and downregulated microRNAs in malignant tumors. One of them, miR‐196b‐5p, showed good overall diagnostic performance. Pathway analysis revealed that downstream targets common to the upregulated microRNAs are implicated in biological processes such as cell proliferation and negative regulation of apoptosis, which may explain the biological features of malignant LCTs and offer options for tailored therapy. Additional studies are needed to determine whether this tissue‐based biomarker is also suitable for assessment in circulation.

## Author contributions statement

JL and AMA conceived, designed and coordinated the study. JL and NTT drafted the manuscript. JL, NTT, FFP, DF, BL, VC and ATM were responsible for the study methodology. KM, KC, MC, CR, MTI, FC, IMF, WA, FM, AOO, CSK, AS, TU, AMA, CJ and RH provided the study cases. TU and AMA undertook critical revision of the draft manuscript. All authors read and approved the final manuscript.

## Supporting information


**Figure S1.** MicroRNA levels in benign and malignant LCTs
**Figure S2.** UMAP (A) and *t*‐SNE (B) analysis of differentially expressed microRNAs in malignant LCTs
**Figure S3.** GATA6 and MKI67 transcript levels in benign versus malignant LCTs
**Table S1.** Clinicopathological features of malignant cases in study cohort
**Table S2.** MicroRNA panel biomarker performance for discriminating malignant from benign LCTs

**Table S3.** Full microRNA list analyzed in profiling (provided as separate Excel file)
**Table S4.** List of significant differentially expressed microRNAs (provided as separate Excel file)
**Table S5.** Ranking of upregulated microRNAs (provided as separate Excel file)
**Table S6.** Ranking of downregulated microRNAs (provided as separate Excel file)
**Table S7.** Target genes for miR‐181c‐5p (provided as separate Excel file)
**Table S8.** Pathways in which target genes for miR‐181c‐5p are involved (provided as separate Excel file)
**Table S9.** Target genes for miR‐182b‐5p (provided as separate Excel file)
**Table S10.** Pathways in which target genes for miR‐182b‐5p are involved (provided as separate Excel file)
**Table S11.** Target genes for miR‐196b‐5p (provided as separate Excel file)
**Table S12.** Pathways in which target genes for miR‐196b‐5p are involved (provided as separate Excel file)

## Data Availability

All data produced in this manuscript are provided in the manuscript and Supplementary materials. The data that support the findings of this study are also available from the corresponding author upon reasonable request. Disclosure: Of note, preliminary results were published as an abstract and presented as a platform session at the 114th USCAP Annual Meeting (Boston, March 2025).
