# Supplementary material for: MicroRNA profiling of testicular Leydig cell tumors identifies a microRNA signature associated with malignancy and miR‐196b‐5p as a potentially useful biomarker
Source: J Pathol. 2025 Oct 28;268(1):54–64. doi: 10.1002/path.6487 (PMC12699239; doi:10.1002/path.6487)
Supplement: Supplementary file 2 — Table S3. Full microRNA list analyzed in profiling (provided as separate Excel file) Table S4. List of significant differentially expressed microRNAs (provided as separate Excel file) Table S5. Ranking of upregulated microRNAs (provided as separate Excel file) Table S6. Ranking of downregulated microRNAs (provided as separate Excel file) Table S7. Target genes for miR‐181c‐5p (provided as separate Excel file) Table S8. Pathways in which target genes for miR‐181c‐5p are involved (provided as separate Excel file) Table S9. Target genes for miR‐182b‐5p (provided as separate Excel file) Table S10. Pathways in which target genes for miR‐182b‐5p are involved (provided as separate Excel file) Table S11. Target genes for miR‐196b‐5p (provided as separate Excel file) Table S12. Pathways in which target genes for miR‐196b‐5p are involved (provided as separate Excel file) [file PATH-268-54-s002.docx]

**MicroRNA profiling of testicular Leydig cell tumors identifies a microRNA signature associated with malignancy and miR-196b-5p as a potentially useful biomarker**

J Lobo, NT Tavares *et al. J Pathol* <https://doi.org/10.1002/path.6487>

**Supplementary Figures S1–S3**

**Supplementary Tables S1 and S2**

**Supplementary Tables S3–S12 are provided as separate Excel files**

**
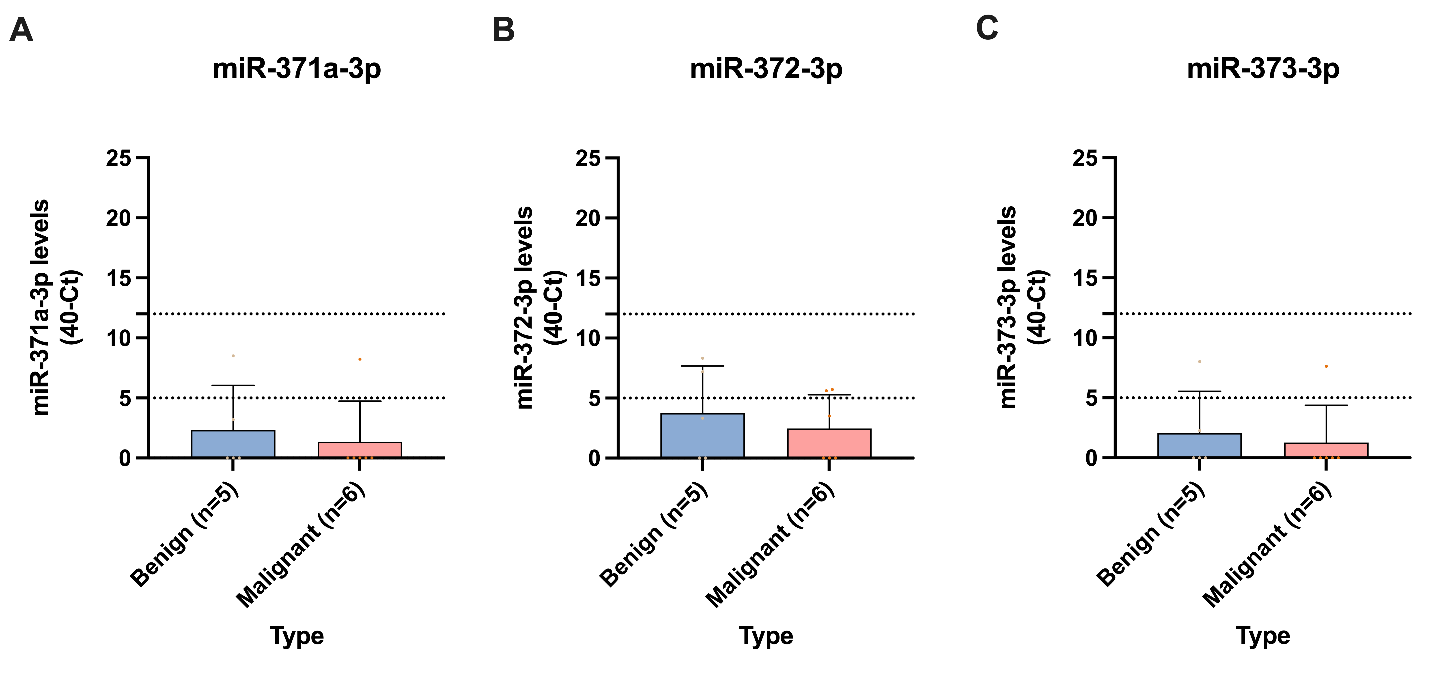
**

**Figure S1.** **MicroRNA levels in benign and malignant LCTs.** MicroRNA levels in benign and malignant LCTs for (A) miR-371a-3p, (B) miR-372-3p, and (C) miR-373-3p. Results are plotted as 40-Ct.


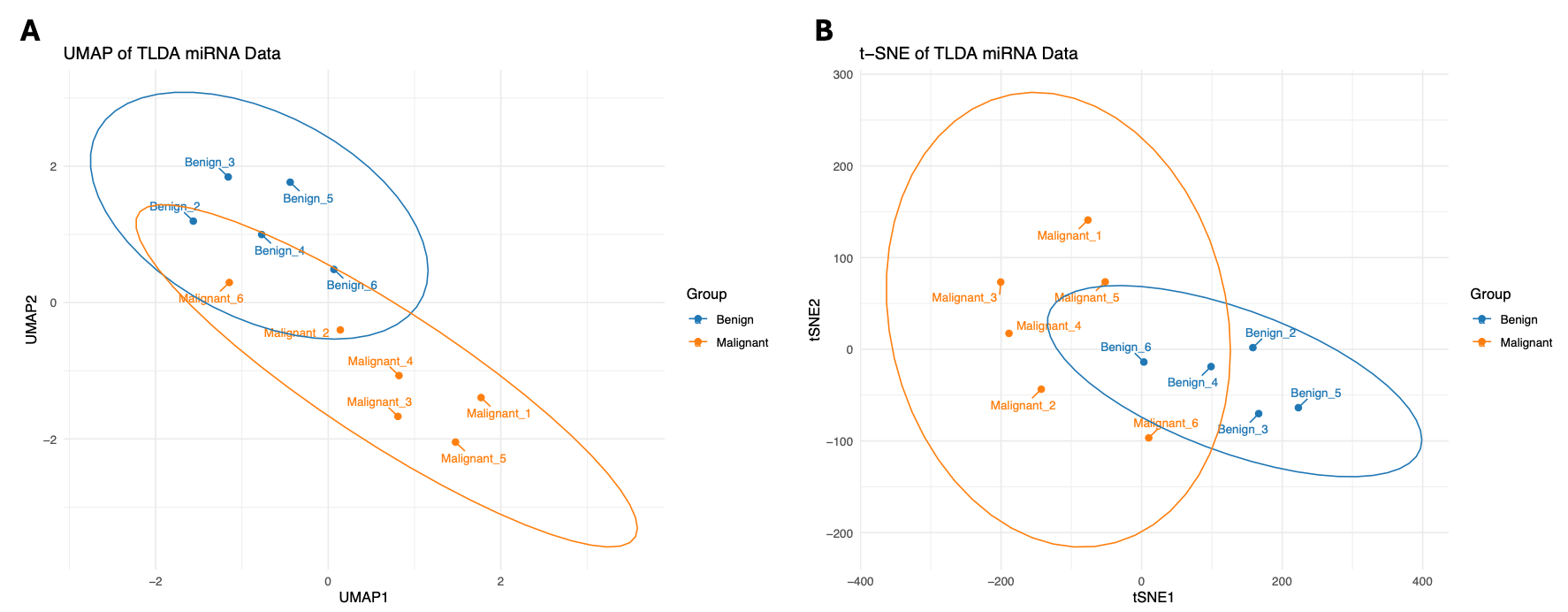


**Figure S2.** **UMAP (A) and *t*-SNE (B) analysis of differentially expressed microRNAs in malignant LCTs.** UMAP (A) and *t*-SNE (B) analysis showing differentially expressed microRNAs reaching statistical significance in malignant LCTs.

**
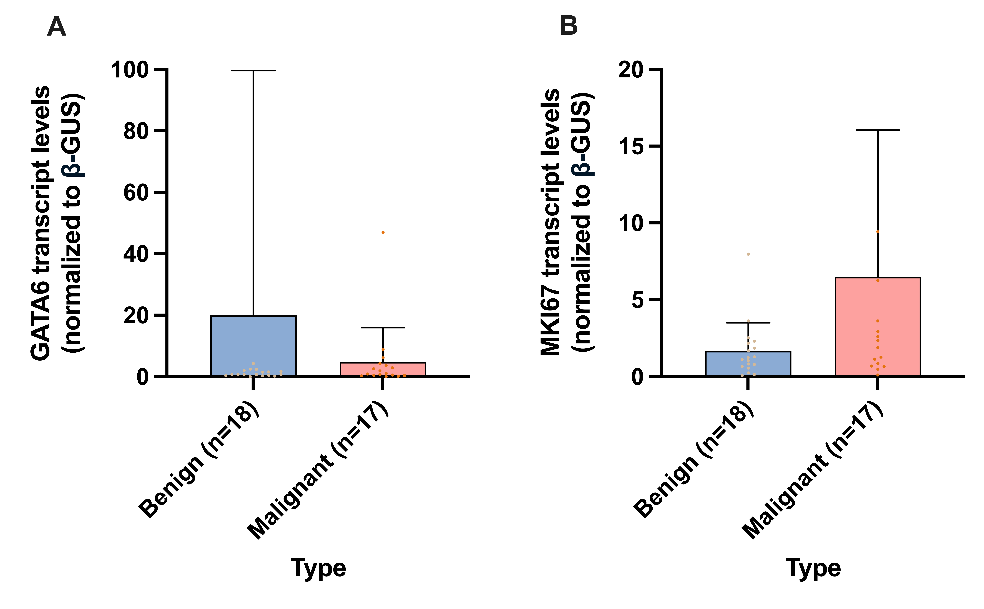
Figure S3.** ***GATA6* and *MKI67* transcript levels in benign *versus* malignant LCTs.** *GATA6* (A) and *MKI67* (B) transcript levels for benign and malignant LCTs (*p* = 0.4428 and *p* = 0.0959, respectively). Results are plotted on a logarithmic scale, normalized to β-glucuronidase (*GUS*) transcript levels.

**Table S1.** Clinicopathological features of malignant cases in study cohort.

| **Case** | **Age (years)** | **Location** |
| --- | --- | --- |
| ML1 | 82 | Metastatic to multiple retroperitoneal LN |
| ML2 | 70 | Metastatic to multiple retroperitoneal LN |
| ML3 | 68 | Primary, with several features of malignancy [extensive LVI, 3 mitosis/10 HPF, atypia, extratesticular extension, >2.5 cm (4 cm)] |
| ML4 | 53 | Metastatic to pelvis, abdominal wall, mesentery, peritoneum, and urachus |
| ML5 | 72 | Metastatic to multiple retroperitoneal LN |
| ML6 | 59 | Metastatic to multiple retroperitoneal LN |
| ML7 | 60 | Metastatic to multiple retroperitoneal LN and lung |
| ML8 | 56 | Primary, with several features of malignancy [17 mitosis/50 HPF, > 2.5 cm (4.7 cm), necrosis, LVI, atypia] |
| ML9 | 66 | Primary, with several features of malignancy [>4 mitosis/ HPF, > 2.5 cm (5 cm), necrosis, infiltrative borders and LVI] and FH-deficiency by immunohistochemistry |
| ML10 | 77 | Metastatic (not otherwise specified) |
| ML11 | 56 | Primary, with several features of malignancy [five mitosis/ HPF, > 2.5 cm (3.1 cm), necrosis, atypia] |
| ML12 | 61 | Metastatic to liver, lung, retroperitoneal LN. Primary tumor with several features of malignancy (>17 mitosis/10 HPF, LVI, infiltrative borders, extratesticular extension) |
| ML13 | 75 | Metastatic to inguinal soft tissue |
| ML14 | 64 | Metastatic to lung and retroperitoneal LN. Primary tumor with several features of malignancy [increased mitotic activity, >2.5 cm (7 cm), necrosis, LVI, extratesticular extension] |
| ML15 | 57 | Primary (metastasizing during follow-up – see below) |
| ML16 | 60 | Metastatic to mediastinum |
| ML17 | 78 | Primary, multifocal, with several features of malignancy [15 mitosis/10 HPF, >2.5 cm (5 cm), infiltrative borders, necrosis, marked atypia, Ki67 50–75%] |

**Table S2.** MicroRNA panel biomarker performance for discriminating malignant from benign LCTs.

| **Panel** | **AUC** | **Sensitivity (%)** | **Specificity (%)** | **Positive predictive value (%)** | **Negative predictive value (%)** | **Accuracy (%)** |
| --- | --- | --- | --- | --- | --- | --- |
| Any positive microRNA | 0.722 | 100 | 44.4 | 63 | 100 | 71.4 |
| 2 positive microRNAs | 0.889 | 100 | 77.8 | 81 | 100 | 89 |
| 3 positive microRNAs | 0.917 | 100 | 83.3 | 85 | 100 | 94.1 |
| 4 positive microRNAs | 0.794 | 58.8 | 100 | 100 | 72 | 80 |
| 5 positive microRNAs | 0.706 | 41.2 | 100 | 100 | 64.3 | 74.1 |
| All positive microRNAs | 0.559 | 11.8 | 100 | 100 | 55 | 57.1 |
